# Molar distalization in orthodontics: a bibliometric analysis

**DOI:** 10.1007/s00784-024-05520-w

**Published:** 2024-01-30

**Authors:** Lin Cheng, Zezhou Feng, Zhaonan Hao, Minmin Si, Rui Yuan, Zhiyuan Feng

**Affiliations:** 1 Shanxi Bethune Hospital, Shanxi Academy of Medical Sciences, Tongji Shanxi Hospital, Third Hospital of Shanxi Medical University, Taiyuan, China; 2 Shanxi Province Key Laboratory of Oral Diseases Prevention and New Materials, School and Hospital of Stomatology, Shanxi Medical University, Taiyuan, China; 3 Department of Orthodontics, Shanxi Provincial People’s Hospital, The Fifth Clinical Medical College of Shanxi Medical University, Taiyuan, China

**Keywords:** Molar distalization, Molar distalisation, Bibliometrics, Orthodontics, Distal movement, Anchorage

## Abstract

**Objectives:**

The study endeavors to undertake a bibliometric analysis on molar distalization, with the objective of illuminating its evolutionary trajectory, current status, and prognosticating future research hotspots and trends.

**Material and methods:**

A comprehensive exploration of the literature on molar distalization was carried out by conducting a search in the Web of Science (WOS) core database of the University of Hong Kong Electronic Library. The search for topic terms employed included “molar distalization,” “molar distalisation,” “move molar distally,” “molar distal movement,” and “molar backwards.” The search results were subsequently subjected to meticulous analysis using CiteSpace software. This analysis encompassed various facets such as the citation count; the geographical distribution of the countries, institutions, and journals responsible for publishing the articles; the distribution of the authors; the utilization of keywords within the articles; and the analysis of references.

**Results:**

A total of 516 articles were included in the analysis. The top 5 countries in terms of the number of published papers were the United States (USA), South Korea, Turkey, Italy, and Germany, and the top 5 institutions in terms of the number of published papers were Kyung Hee University, A.T. Still University of Health Sciences, Catholic University of Korea, Seoul St. Mary’s Hospital, and Universidade de Sao Paulo. The top 5 authors in terms of the number of published papers were Park, Kook, Bayome, Janson, and Lee. There was little cooperation overall. The top 3 journals in terms of the most published related articles were all orthodontic-related journals. After molar distalization and anchorage, the most frequently used keywords were distalization, movement, and pendulum appliance. Kinzinger GSM is the most frequently cited author in references, and one of his articles also has the highest centrality score in references.

**Conclusions:**

As the tides of time shift and scholars display an ever-growing dedication to unraveling the intricacies of this therapeutic modality, the realm of molar distalization has undergone notable advancements in technology. Initially, the traditional appliance suffered from aesthetic drawbacks and discomfort. However, contemporary iterations of the appliance have transcended these limitations, boasting enhanced elegance and convenience while concurrently elevating their efficacy. Nevertheless, limitations of current appliances, including their durability and propensity for recurrence post-treatment, continue to necessitate further advancement. Hence, the ongoing scientific inquiry aims to delve deeper into refining treatment modalities and fabricating cutting-edge appliances within this realm.

Clinical relevance.

This study holds the potential to significantly enhance the ability of orthodontists to devise treatment protocols and offer state-of-the-art clinical recommendations, thereby empowering them to deliver advanced and refined orthodontic interventions.

## Introduction

The technique of molar distalization primarily finds its application in cases of mild to moderate dental crowding [1]. This approach is most apt for circumstances where there is a reluctance for tooth extraction despite the presence of dental overcrowding. Moreover, the distalization of the maxillary molars is implemented to rectify Angle Class II malocclusions [2, 3], whereas the distalization of mandibular molars alleviates Angle Class III malocclusion. Simultaneous distalization of both maxillary and mandibular molars proffers a remedy for both maxillary and mandibular prognathism. At present, the research on molar distalization mainly focuses on maxillary molar distalization to solve the crowding of maxillary dentition or Class II malocclusion [4].

With the development of time, the main effective methods of molar distalization have changed significantly. Initially, extraoral appliances such as headgear [5] and extraoral arches are used, an approach that is not only unaesthetic but also uncomfortable for the patient [6, 7]. In order to solve this problem, intraoral appliances such as pendulum [8] and frog appliances have been developed. This kind of appliance is relatively better and more efficient, and patients will feel more comfortable. The disadvantage is that it will cause loss of anchorage [9]. At present, the treatment methods are more diversified, such as the combination of intraoral instruments and micro-implants, the promotion of clear aligners, and the 3D printing technology for the manufacture of orthodontic appliances. The combination of intraoral fixed appliance and micro-implants [10] not only has good therapeutic effect but also can significantly reduce the loss of anchorage [11–13]. Clear aligners are also effective and aesthetically pleasing in the treatment of molar distalization [14, 15]. The application of 3D printing technology in the field of orthodontics makes the production of orthodontic appliances more precise and more suitable for patients, and the treatment effect is also improved. These advances in technology gives the orthodontist a wider range of treatment options and the patient more options.

Recent years, there has been a growing number of clinical studies and reviews related to molar distalization. However, according to our search of the relevant literature, bibliometric analysis and bibliometric mapping have not been used to analyze the literature production of molar distalization [16]. Using bibliometrics, we examined the dynamics and trend patterns of literature production and identified literature types and the most prolific authors, institutions, and countries, as well as the common collaborations among them [16]. At the same time, it also includes the analysis of keywords and references in the literature and the discussion of current research hotspots. Citation analysis is a commonly used method in bibliometric research to assess the impact of publications [17]. CiteSpace represents a widely recognized software for bibliometric analysis, facilitating the visualization of pertinent literature data and examination of research trajectories within a given sphere. In contrast to conventional reviews, bibliometric approaches enable the expeditious and precise identification of prominent research avenues and salient information, thereby providing guidance for future investigative focal points [18].

The objective of this investigation was to perform a comprehensive bibliometric evaluation of the corpus of literature pertaining to the distalization of impacted molars. Through this study, we aim to elucidate the progression and maturation of the associated literature within this domain; underscore the contributions and collaborative relationships prevalent among authors, nations, and institutions; pinpoint journals of authority; scrutinize influential citations along with their authors; and delineate prevailing research focal points as well as prospective research trajectories via keyword analysis.

## Methods

### Database

Bibliometric research predominantly relies on the Web of Science (WOS) core database of the University of Hong Kong Electronic Library as the primary data source for retrieval [19]. This database, established by the American Institute for Scientific Information in 1957, encompasses a vast repository of scholarly articles and associated citation data from over 8000 influential journals [19]. Emanating as a pivotal instrument for citation retrieval, the Web of Science (WOS) core database assumes a paramount role in the realm of metrology research and scientific evaluation, rendering it an indispensable resource of utmost significance.

### Search strategy

In this study, a “topic” search strategy was adopted, the Web of Science Core Collection at the library of Hong Kong University was searched using “((((TS = (molar distalization)) OR TS = (molar distalisation)) OR TS = (move molar distally)) OR TS = (molar distal movement)) OR TS = (molar backwards)” as the search terms. The document types included article and review article. There was no restriction on the publication time of the articles, and the last retrieval date was September 8, 2023.

### Data screening and collection

**Fig. 1:**
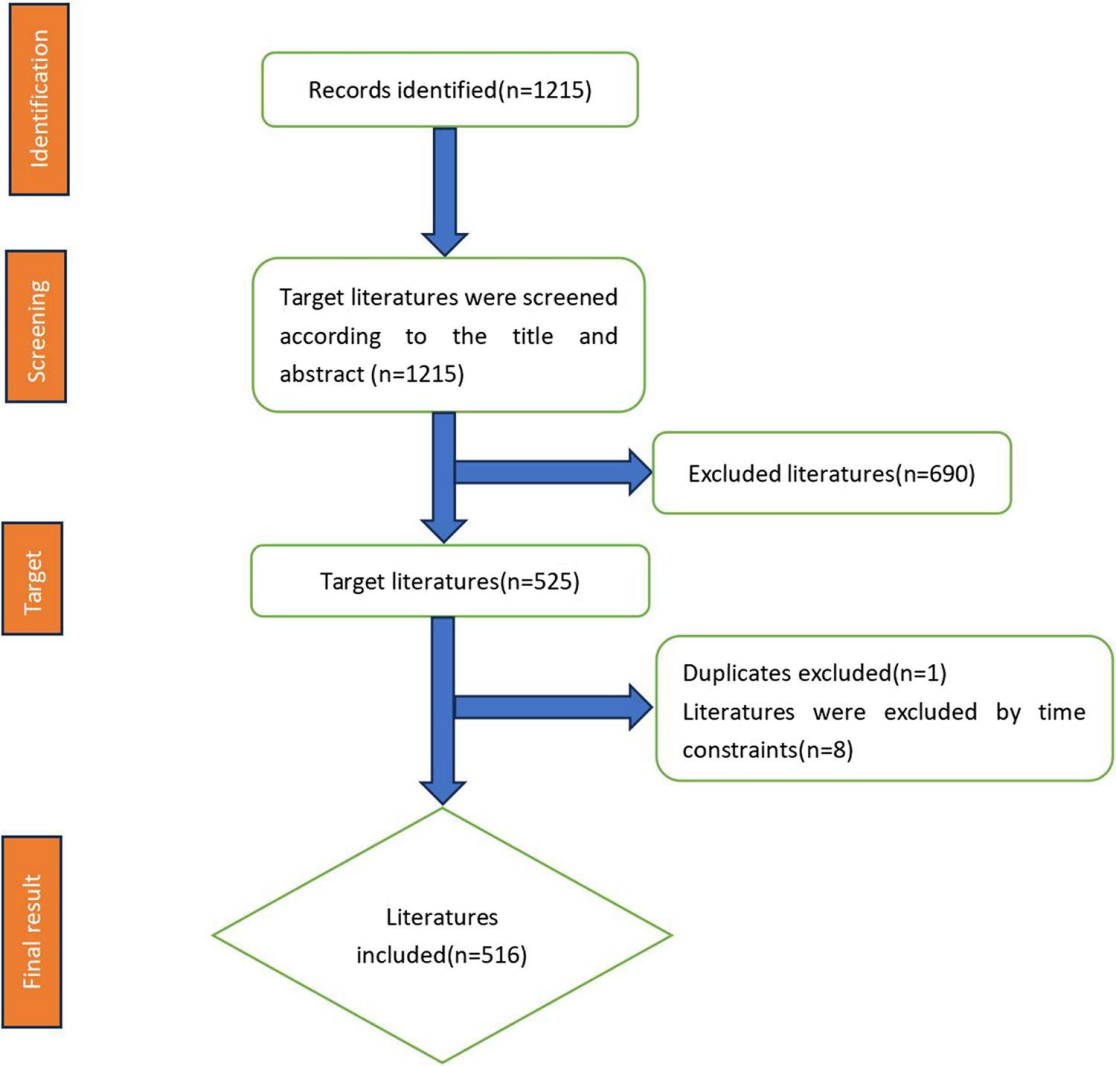
Data screening

In the process of data screening, a total of 525 relevant literatures were retrieved, among which one was a duplicate literature. Then, the retrieval time span was set to 1993 to 2023, and the relevant literature data was obtained to 516 (see Fig. 1). Upon the successful completion of the exhaustive literature search, the obtained search results and their corresponding cited references were exported as plain-text files, serving as the foundation for subsequent analysis. To undertake this analysis, the CiteSpace software was employed to unravel the intricate complexities within the sourced material. The analysis encompassed various facets, including the annual distribution of the articles; the distribution of institutions and countries associated with said articles; the allocation of authorship, collaborative efforts between countries, institutions, and authors; the distribution of articles across scholarly journals; the usage patterns of keywords within the articles; and analysis of references.

### Statistical analysis

The crux of this study revolved around the utilization of numerical values and corresponding percentages to portray the statistical indicators. Pertinently, no comparative analyses were undertaken, thereby obviating the necessity for establishing a test level.

## Results

### General information

**Fig. 2:**
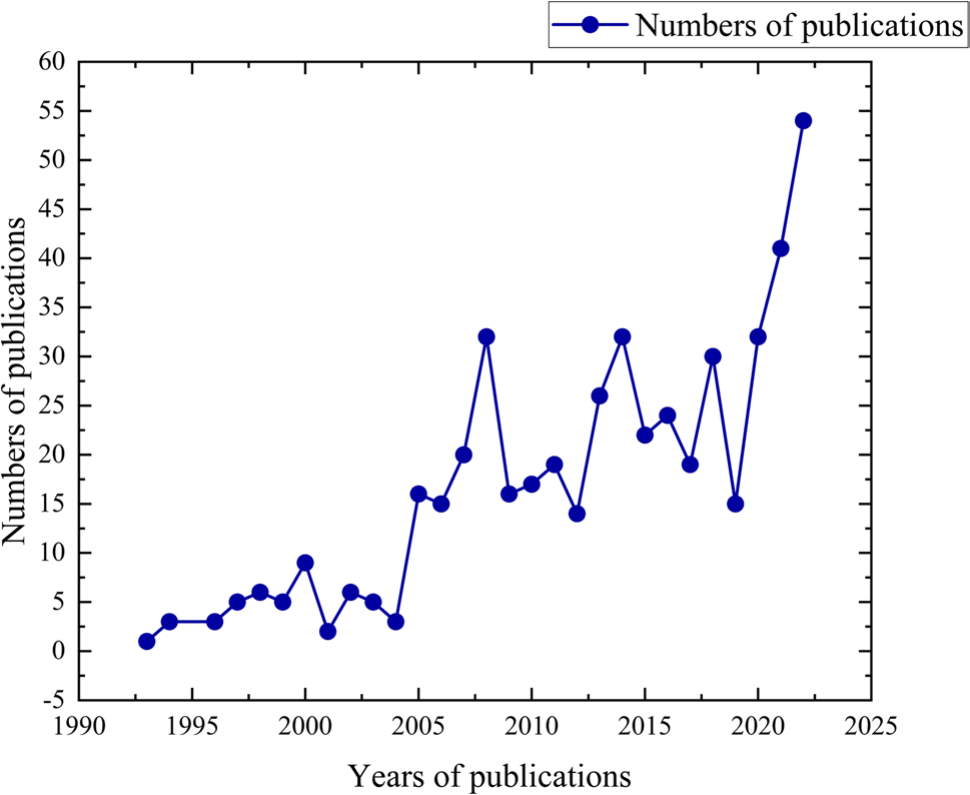
Annual changes in the number of articles published

A total of 516 articles were included in the analysis, and these articles were cited 6263 times, with an average of 12.14 citations per article. Among these articles, there were 481 original articles, 34 reviews, and 1 proceeding papers (see Table 1). The number of published papers per year did not change much before 2005 but showed an overall growth trend after 2005, and the number of published papers reached its peak in 2022 (see Fig. 2).

**Table 1:** Article type

Article type	Records	% of 516
Article	481	93.22
Review	34	6.59
Proceedings paper	1	0.19

### Countries and institutions

**Fig. 3:**
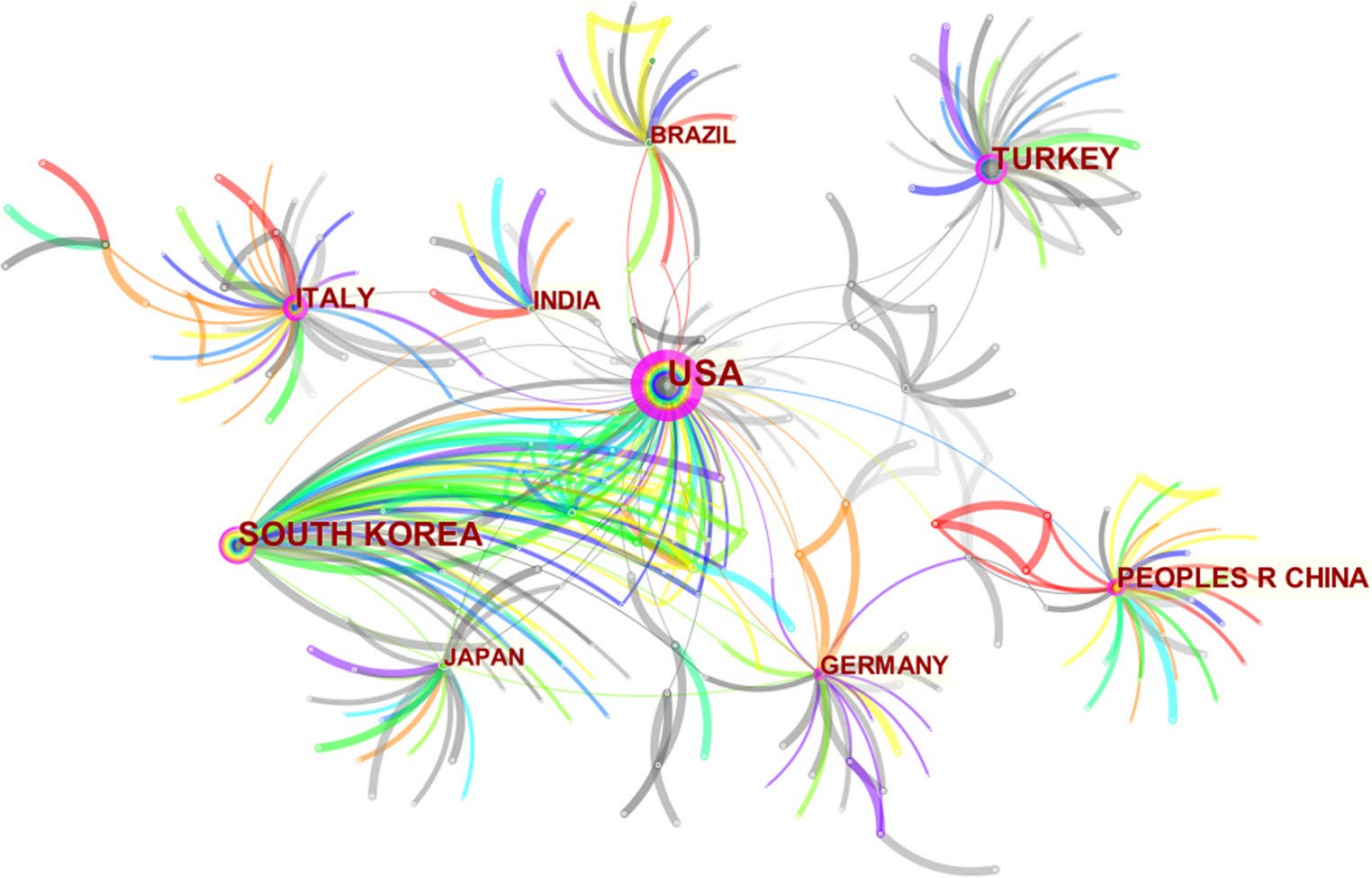
Co-occurrence map of countries

**Fig. 4:**
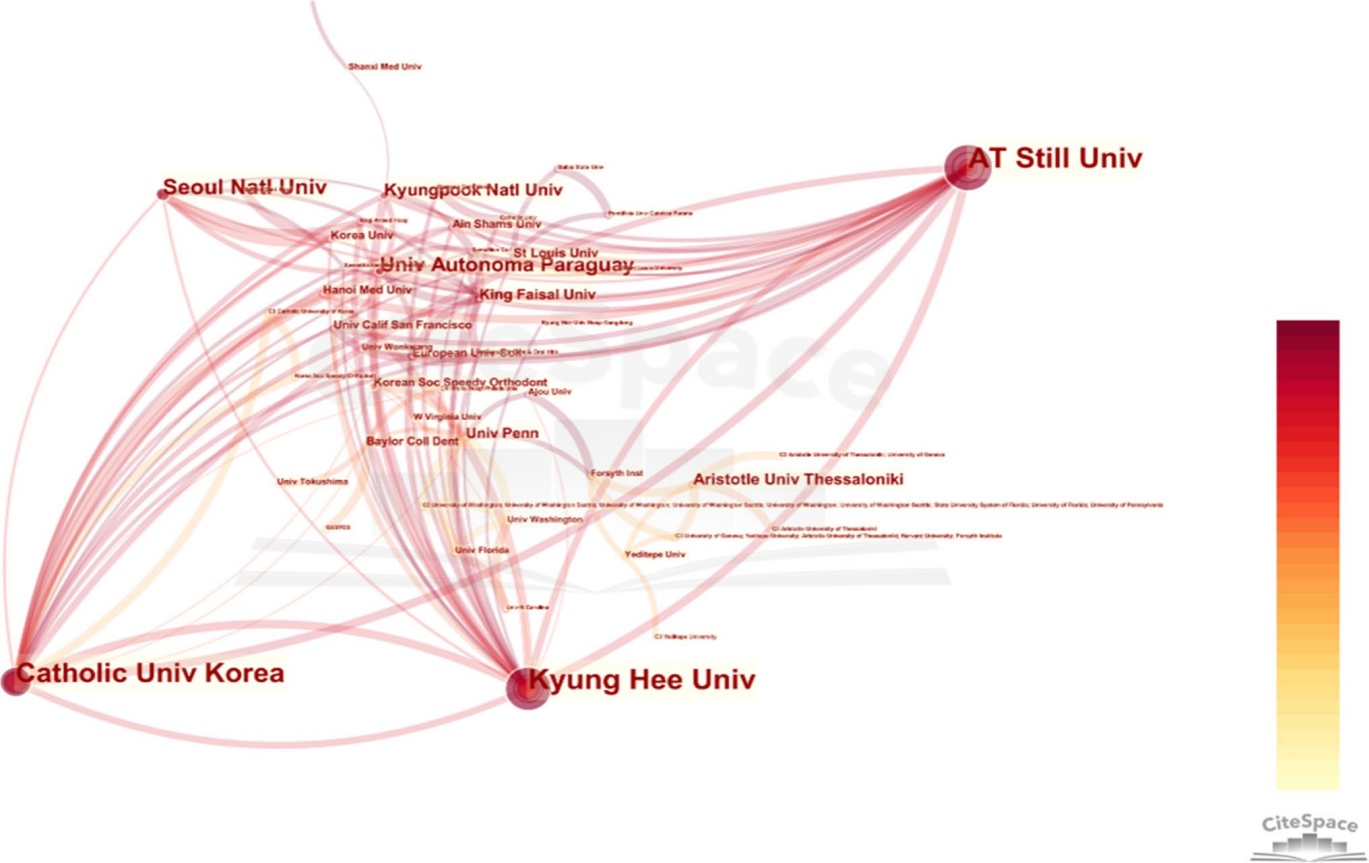
Co-occurrence map of research institutions

The analysis of country and institutional data within the literature sources was conducted employing the advanced CiteSpace software, resulting in the generation of a visually captivating visualization map. Within this map, a total of 52 countries were identified as network nodes, as depicted in the accompanying (see Fig. 3). Remarkably, these nodes were interconnected a staggering 408 times, denoting instances where two countries were simultaneously referenced within the same document. These findings provide valuable insights into the intricate web of collaborative endeavors among countries within the field under investigation. Additionally, the institutional visualization map (see Fig. 4) exhibited a rich tapestry of 451 network nodes. Remarkably, each node symbolized the active participation of a distinct research institution in the specific domain under consideration. Notably, these institutions collectively engaged in a remarkable total of 487 collaborative endeavors, as substantiated by the interconnectedness observed within the visual depiction.

The statistical analysis further revealed the leading nations in terms of publication output, with the USA, South Korea, Turkey, Italy, and Germany emerging as the top five countries, as illustrated in the accompanying Table 2. To gauge the extent of collaborative efforts between nations, the centrality score served as a paramount indicator. Notably, the USA, Italy, Slovakia, Saudi Arabia, and the Czech Republic emerged as the top five countries in terms of cooperation, as depicted in the aforementioned Table 3. Turning our attention to institutions, the top five entities in terms of publication volume were Kyung Hee University, A.T. Still University of Health Sciences, Catholic University of Korea, Seoul St. Mary's Hospital, and Universidade De Sao Paulo, as outlined in the Table 4. Remarkably, Kyung Hee University was the sole institution to exhibit a centrality score > 0.01 (see Table 5), thus suggesting a heightened level of collaborative engagement.

**Table 2:** Top 10 countries by posts

Rank	Countries	Frequency
1	USA	114
2	South Korea	78
3	Turkey	73
4	Italy	45
5	Germany	42
6	China	40
7	Brazil	36
8	India	34
9	Japan	34
10	Switzerland	14

**Table 3:** Top 10 countries by centrality

Rank	Countries	Centrality
1	USA	0.72
2	Italy	0.35
3	Slovakia	0.16
4	Saudi Arabia	0.11
5	Czech Republic	0.11
6	China	0.10
7	South Korea	0.08
8	Germany	0.07
9	Brazil	0.06
10	India	0.06

**Table 4:** Top 10 institutions by publishing volume

Rank	Institutions	Frequency
1	Kyung Hee University	40
2	A.T. Still University of Health Sciences	35
3	Catholic University of Korea	34
4	Seoul St. Mary’s Hosptial	33
5	Universidade De Sao Paulo	19
6	Universidad Autonoma del Paraguay	12
7	Seoul National University	12
8	Baskent University	11
9	Egyptian Knowledge Bank	9
10	Saveetha Dental college	8

**Table 5:** Top 5 institutions by centrality

Rank	Institutions	Centrality
1	Kyung Hee University	0.03
2	Egyptian Knowledge Bank (EKB)	0.01
3	A.T. Still University of Health Sciences	0
4	Catholic University of Korea	0
5	Seoul St. Mary’s Hospital	0

### Authors

**Fig. 5:**
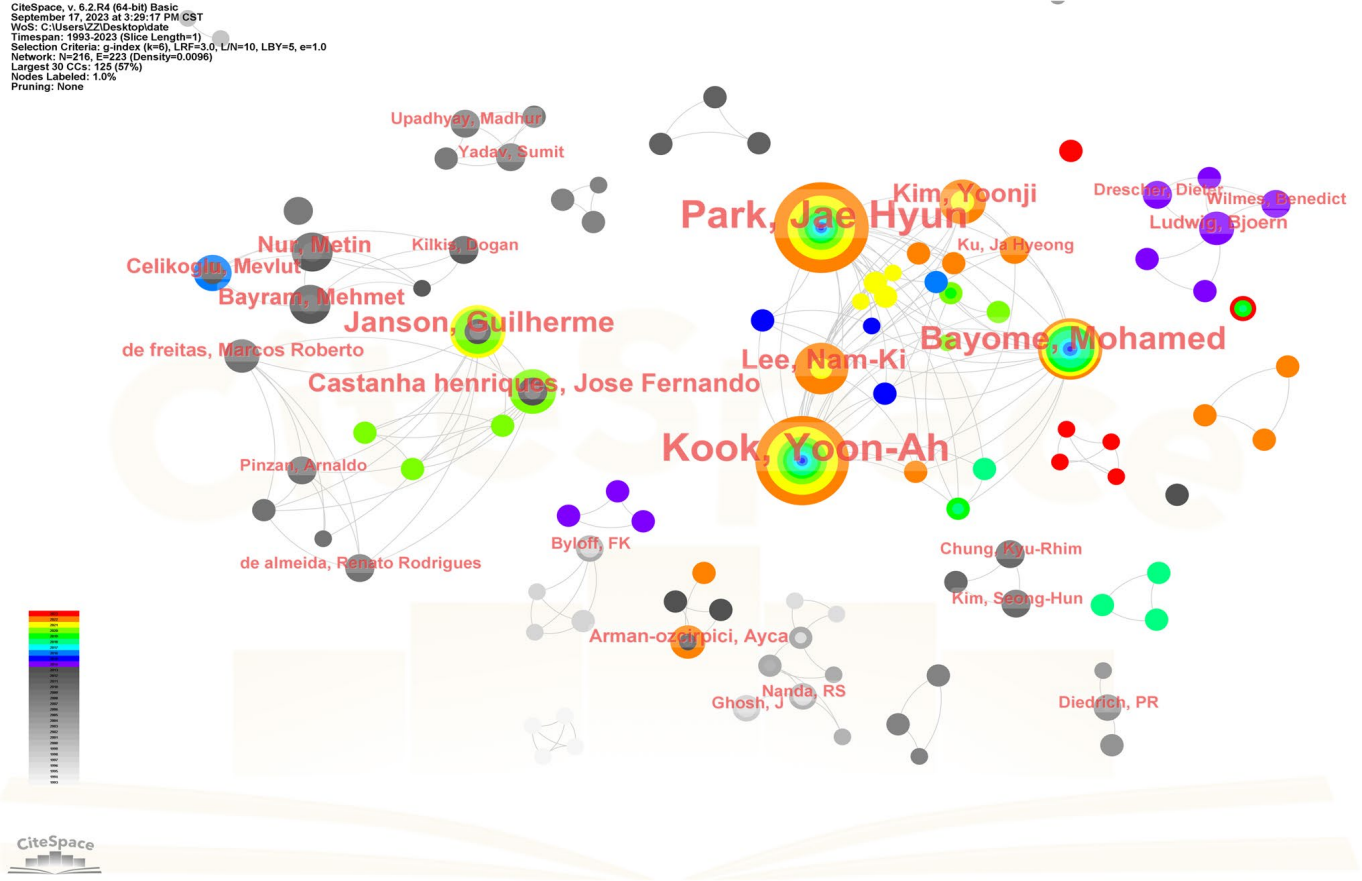
Author co-authored visualization map

**Fig. 6:**
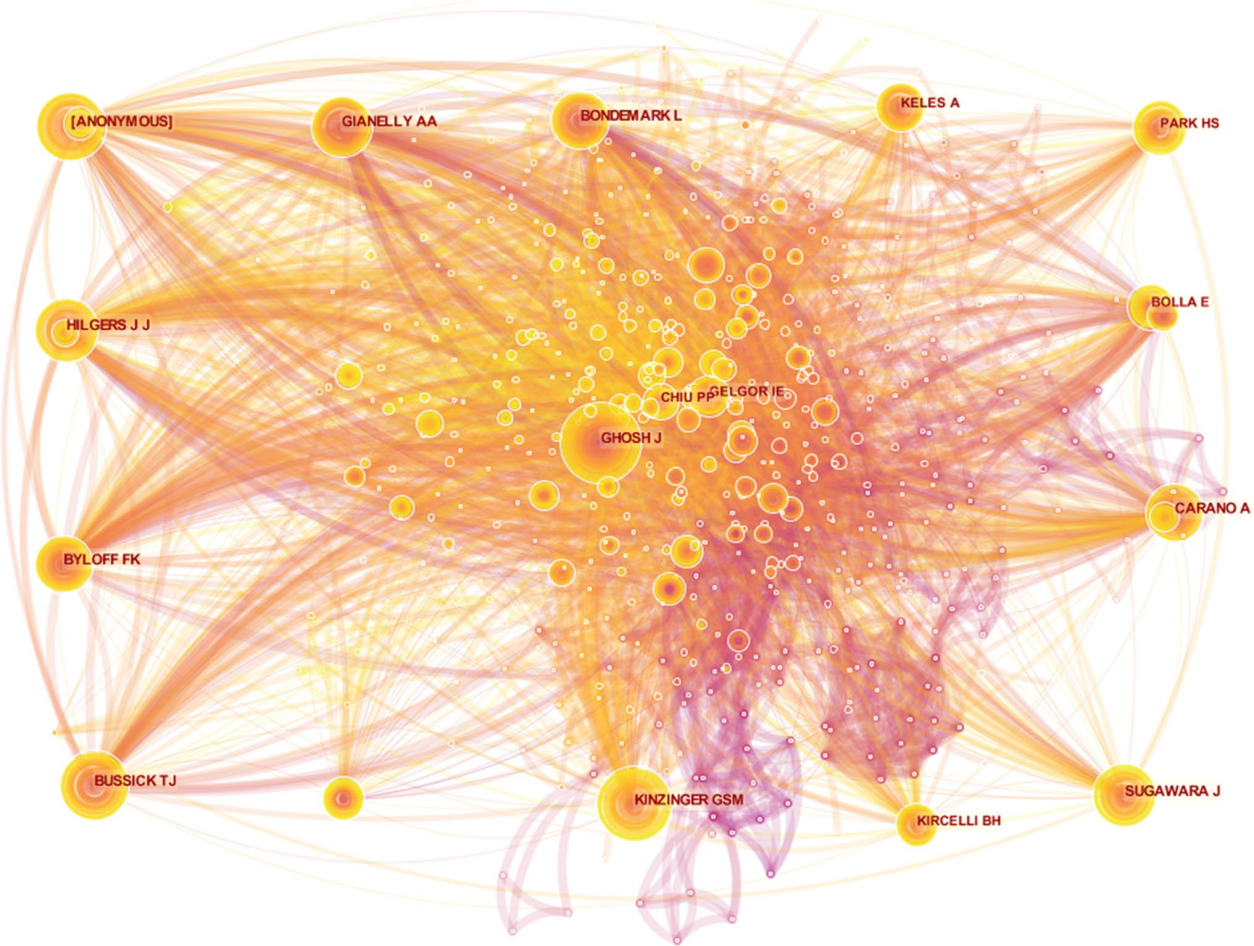
Author co-citation visualization map

The analysis of author information was conducted employing the sophisticated CiteSpace software. Remarkably, the findings unveiled the preeminent contributors in terms of article publications, namely Park Jae Hyun, Kook Yoon-Ah, Bayome Mohamed, Janson Guilherme, and Lee Nam-Ki (refer to Table 6). As discerned from the author cooperation visualization map, a certain degree of collaboration was observed among select authors; however, this collaboration appeared to be somewhat dispersed, primarily constrained within the confines of the same research institution or team (refer to Fig. 5). This observation aligns with the modest centrality scores assigned to these authors, all of them had centrality scores of less than 0.01, with the exception of Park Jae Hyun, who had a centrality score of 0.01. Notably, the top five co-cited authors comprised Hilgers JJ, Kinzinger GSM, Ghosh J, Gianelly AA, and [Anonymous] (refer to Table 7). Of particular significance, Bussick TJ emerged as the author boasting the highest co-citation centrality score (refer to Table 8). Evident from the co-citation visualization map, the interconnections among the cited studies were considerably widespread, implying a close linkage between these scholarly endeavors (refer to Fig. 6).

**Table 6:** Top 10 authors by number of publications

Rank	Authors	Frequency
1	Park, Jae Hyun	32
2	Kook, Yoon-Ah	31
3	Bayome, Mohamed	17
4	Janson, Guilherme	12
5	Lee, Nam-Ki	10
6	Castanha henriques, Jose Fernando	8
7	Kim, Yoonji	8
8	Bayram, Mehmet	6
9	Nur, Metin	6
10	Celikoglu, Mevlut	5

**Table 7:** Top 10 authors with total citations

Rank	Authors	Frequency
1	Hilgers JJ	142
2	Kinzinger GSM	137
3	Ghosh J	130
4	Gianelly AA	121
5	[Anonymous]	118
6	Byloff FK	115
7	Bussick TJ	111
8	Carano A	110
9	Bondemark L	88
10	Park HS	82

**Table 8:** Top 10 centrally cited authors

Rank	Authors	Centrality
1	Bussick TJ	0.19
2	Gianelly AA	0.17
3	Kinzinger GSM	0.15
4	Park HS	0.14
5	[Anonymous]	0.13
6	Bondemark L	0.13
7	Carano A	0.12
8	Keles A	0.1
9	Gelgor IE	0.09
10	Ghosh J	0.08

### Journals

**Fig. 7:**
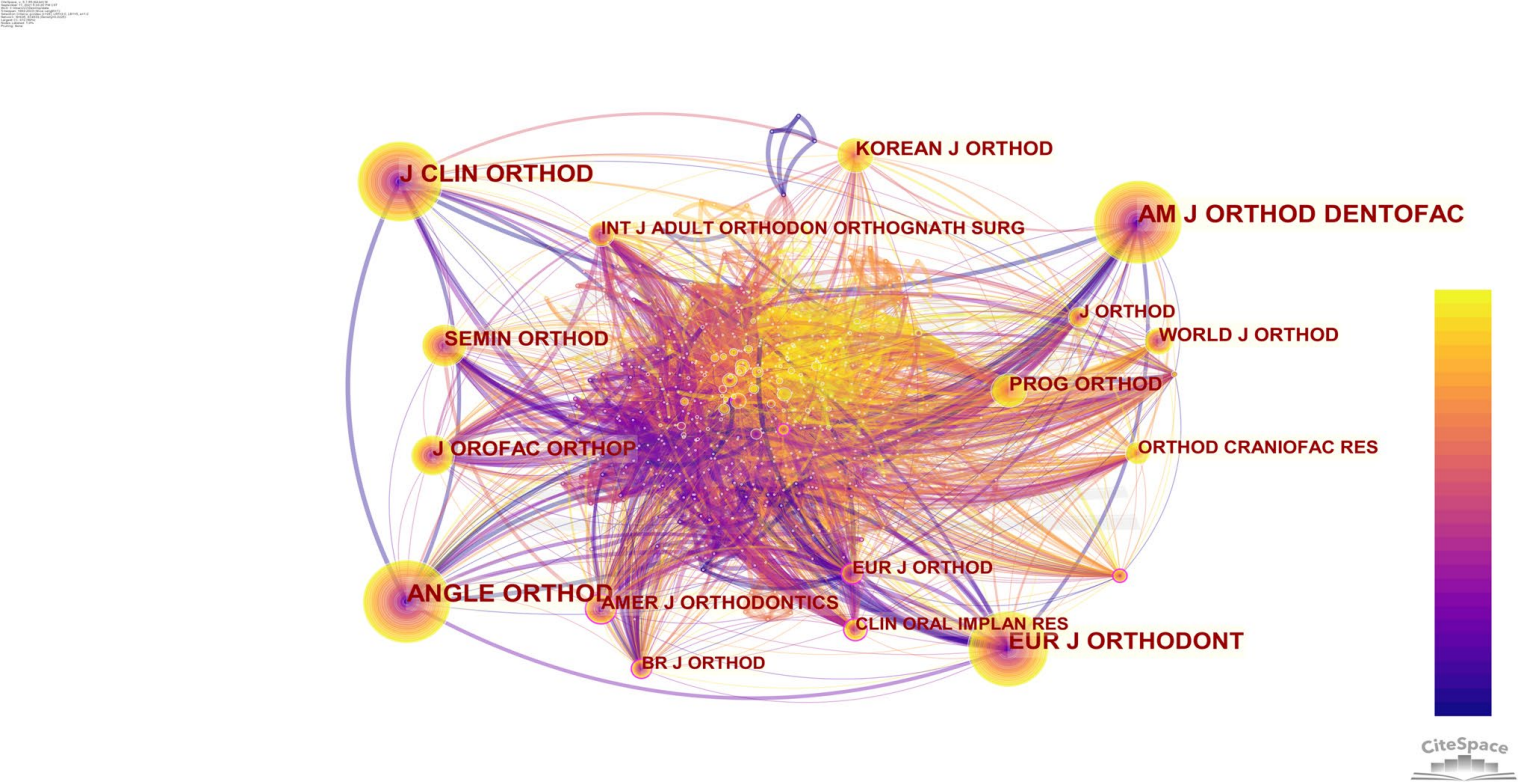
Cluster graph of cited journals

The eminent journals that garnered the highest number of citations encompassed the American Journal of Orthodontics and Dentofacial Orthopedics, Angle Orthodontist, Journal of Clinical Orthodontics, European Journal of Orthodontics, and Journal of Orofacial Orthopedics (see Table 9). Furthermore, the journals exhibiting a notable citation centrality value predominantly included the American Journal of Orthodontics, British Journal of Orthodontics, Contemporary Orthodontics, European Journal of Orthodontics, and Clinical Oral Implants Research (see Table 10); this indicates that there is some cooperation and exchange between these journals (see Fig. 7). Whether classified based on frequency or centrality, these journals consistently upheld their authority as leading sources in the field of orthodontics.

**Table 9:** Top 5 cited journals by frequency

Rank	Cited journals	Frequency
1	AM J ORTHOD DENTOFAC	498
2	ANGLE ORTHOD	477
3	J CLIN ORTHOD	380
4	EUR J ORTHODONT	340
5	J OROFAC ORTHOP	163

**Table 10:** Top 5 cited journals by centrality

Rank	Cited journals	Centrality
1	AMER J ORTHODONTICS	0.15
2	BR J ORTHOD	0.13
3	CONT ORTHODONTICS	0.11
4	EUR J ORTHOD	0.11
5	CLIN ORAL IMPLAN RES	0.10

**Fig. 8:**
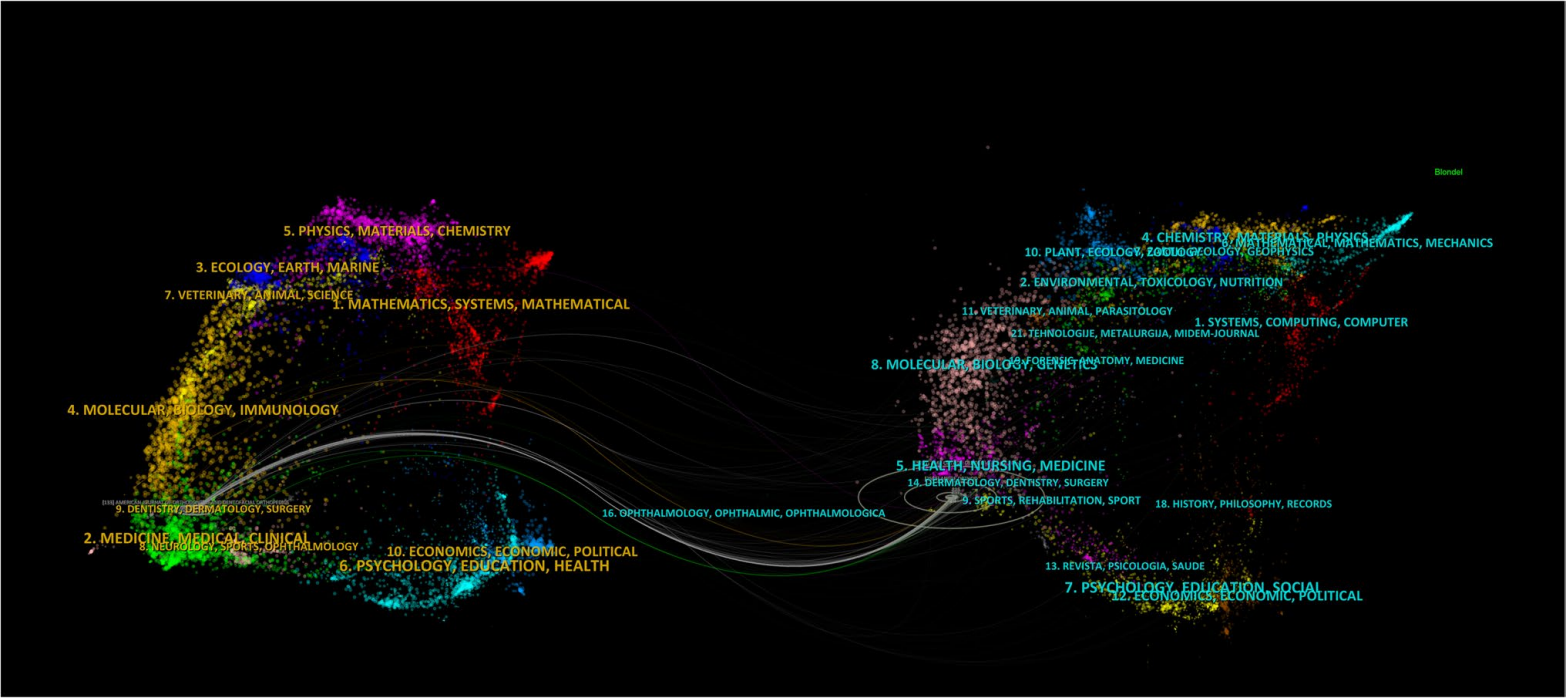
Dual-map of journals. Annotation: Within the presented figure, the cluster positioned on the left signifies the group of journals engaging in citations, whereas the cluster on the right embodies the collection of journals being cited. The citation line, depicted as a prominent curve, serves as a visual representation of the connections between these clusters. Notably, the elongation of the vertical axis within the ellipses correlates with the quantity of papers published within a given journal, while the extension of the horizontal axis reflects the breadth of authors contributing to said publications

CiteSpace’s dual-map of journals was used to cluster and overlay the journals of the samples (see Fig. 8). The citing journals on the left are mainly concentrated in the fields of dentistry, dermatology, and surgery. The cited journals on the right are mainly concentrated in the fields of dentistry, dermatology, and surgery, followed by sports, rehabilitation, sport, health, nutrition and medicine, and so on. Overall, molar distalization has the characteristics of spanning the field of oral medicine and the field of human health nutrition.

### Keywords

**Fig. 9:**
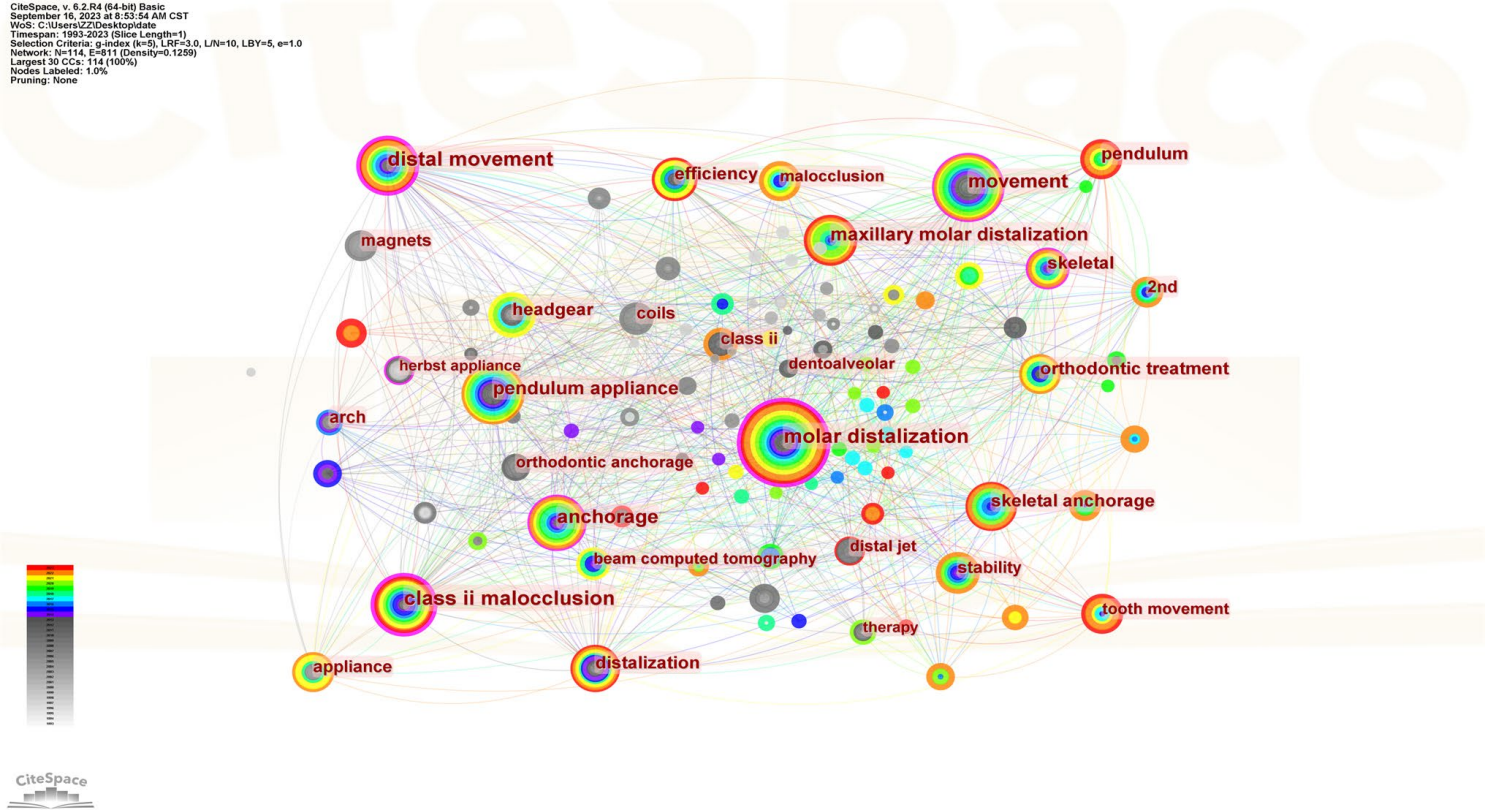
Keyword co-occurrence graph

**Fig. 10:**
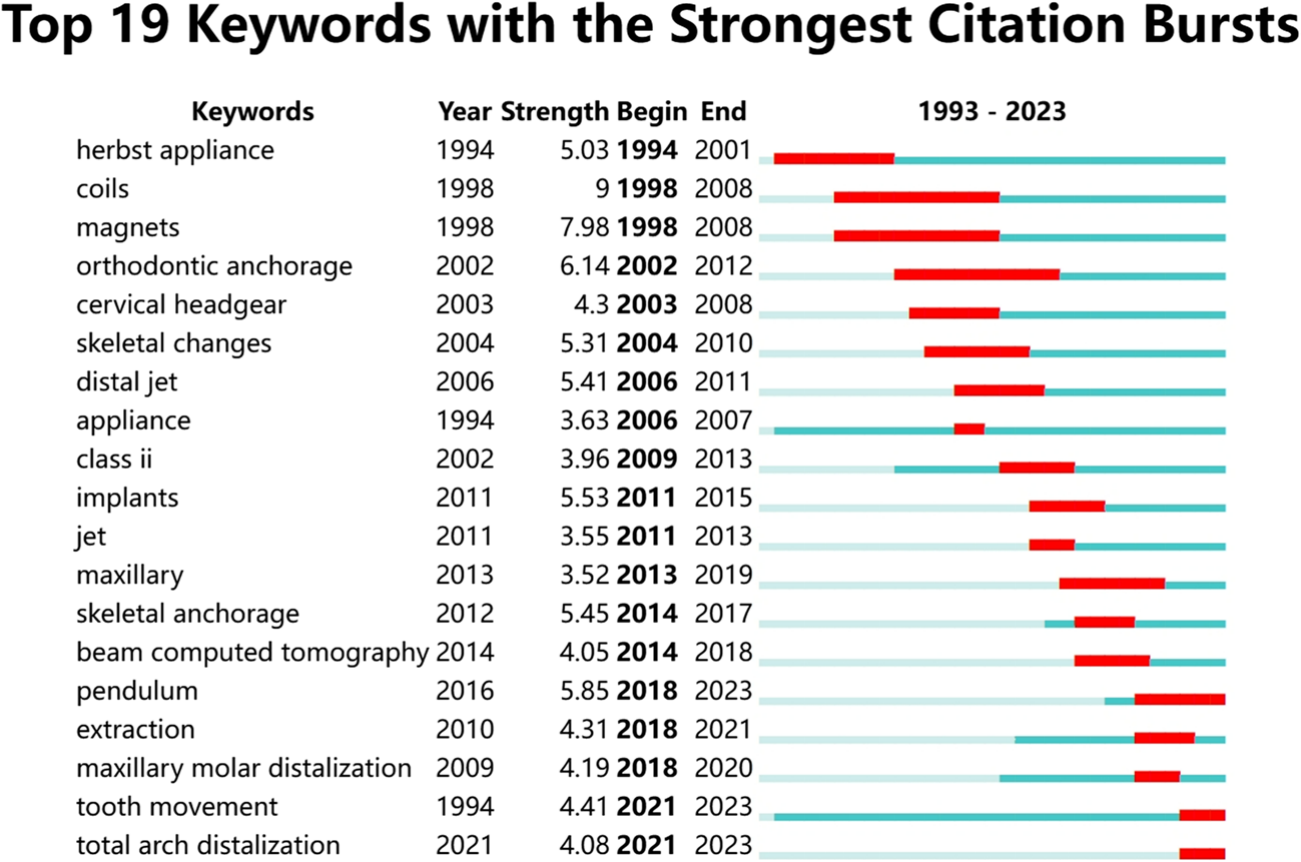
Burst graph of keywords. Annotation: Among the multitude of keywords examined, a notable selection of 19 emerged distinguished by their significant citation bursts. These keywords exhibited pronounced peaks denoted by red lines, symbolizing the years when they were prominently employed. Conversely, green lines signify periods within the timeframe from 1993 to 2023 when these keywords were less frequently utilized

**Fig. 11:**
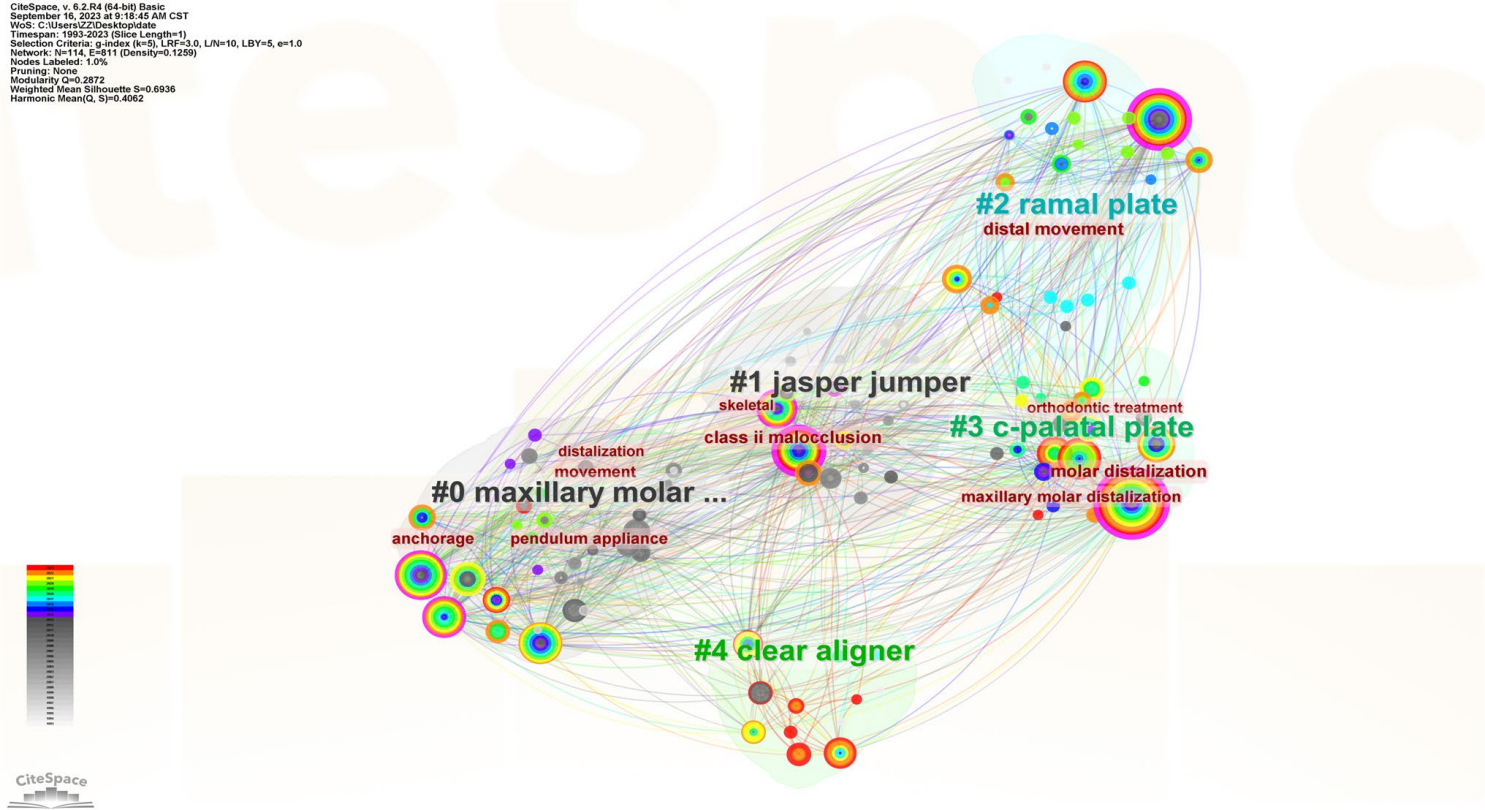
Cluster graph of keywords

**Fig. 12:**
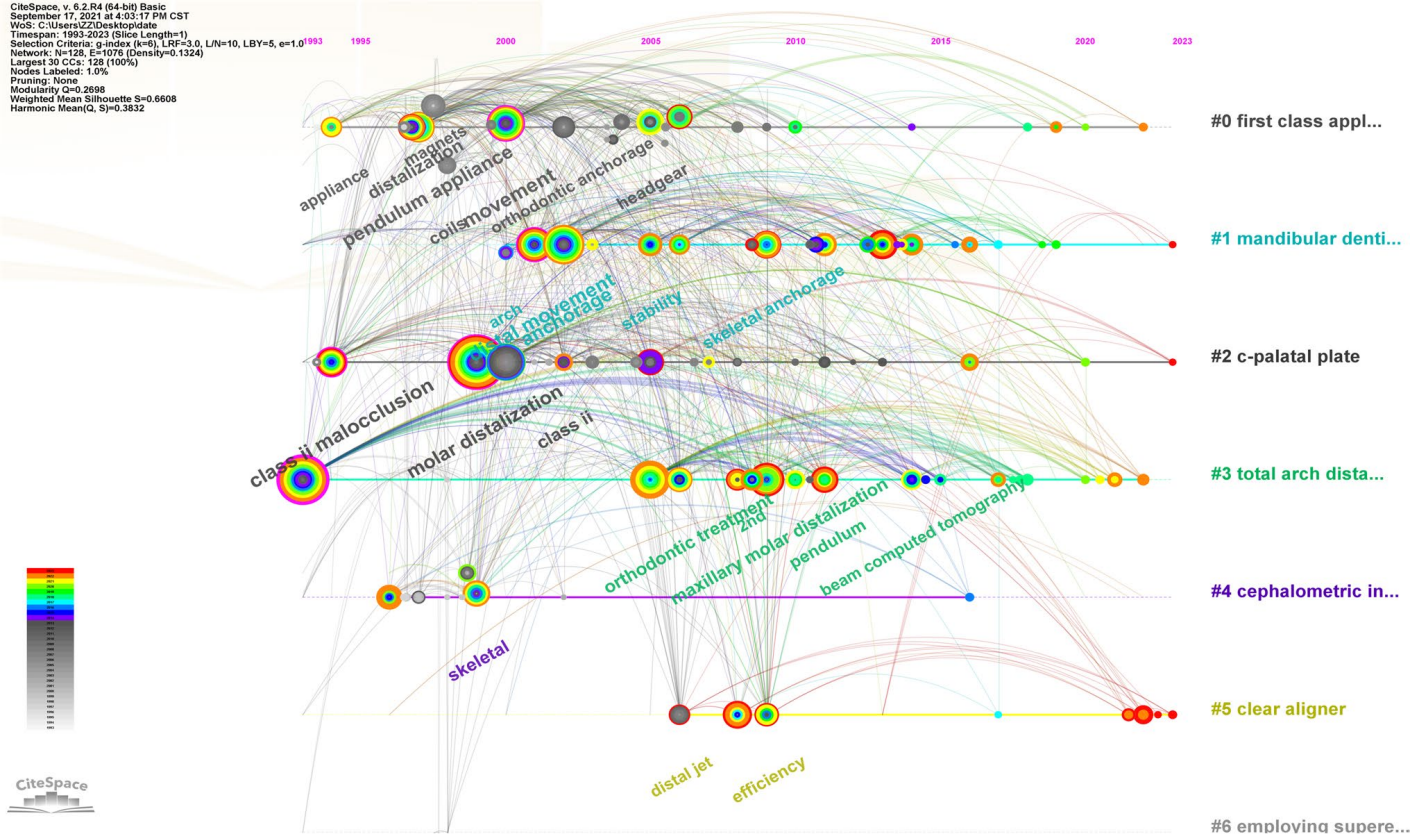
Timeline cluster graph of keywords

CiteSpace software was used to generate a keyword co-occurrence map (see Fig. 9). There were 114 nodes in the figure; that is, in the 516 articles, 114 keywords were used (see Fig. 9). There were 811 connections between the nodes in the graph; that is, 2 of the keywords appeared 811 times in a document at the same time (see Fig. 9). The most frequently used terms were molar distalization and movement (see Table 11). The keywords with the highest centrality scores were class II malocclusion and molar distalization, and other keywords with high scores included distal movement, anchorage, and movement (see Table 12). CiteSpace was also used to conduct a burst analysis of the keywords with a high frequency (see Fig. 10), and the results showed that the use of hot keywords changed over time (Figs. 11 and 12).

**Table 11:** Top 5 keywords by frequency

Rank	Keywords	Frequency
1	Molar distalization	130
2	Movement	68
3	Class II malocclusion	66
4	Distal movement	66
5	Pendulum appliance	65

**Table 12:** Top 5 keywords by centrality

Rank	Keywords	Centrality
1	Class II malocclusion	0.31
2	Molar distalization	0.23
3	Distal movement	0.20
4	Anchorage	0.12
5	Movement	0.11

### Reference analysis

The data presented in Table 13 and Table 14 unequivocally establish the preeminent standing of the author Kinzinger GSM within the scholarly landscape. Not only does Kinzinger GSM command the highest frequency of citation, but his articles also exhibit the most pronounced article centrality scores. These findings eloquently illuminate the pivotal and indispensable role played by Kinzinger GSM in shaping and advancing the field of study under investigation.

**Table 13:** Top 5 references by cited frequency

Rank	Citation frequency	Year of publication	Author	Title
1	26	2004	Kinzinger GSM	Efficiency of a pendulum appliance for molar distalization related to second and third molar eruption stage [20]
2	19	2004	Gelgor IE	Intraosseous screw-supported upper molar distalization [21]
3	18	2020	Bechtold TE	Long-term stability of miniscrew anchored maxillary molar distalization in Class II treatment [11]
4	17	2013	Sar C	Comparison of two implant-supported molar distalization systems [22]
5	17	2007	Escobar SA	Distalization of maxillary molars with the bone-supported pendulum: a clinical study [23]

**Table 14:** Top 5 references by cited centrality score

Rank	Centrality score	Year of publication	Author	Title
1	0.34	2009	Kinzinger GSM	Efficiency of a skeletonized distal jet appliance supported by miniscrew anchorage for noncompliance maxillary molar distalization [24]
2	0.22	2006	Kircelli BH	Maxillary molar distalization with a bone-anchored pendulum appliance [25]
3	0.21	2004	Kinzinger GSM	Efficiency of a pendulum appliance for molar distalization related to second and third molar eruption stage [20]
4	0.21	2013	Sar C	Comparison of two implant-supported molar distalization systems [22]
5	0.18	2007	Escobar SA	Distalization of maxillary molars with the bone-supported pendulum: a clinical study [23]

## Discussion

This study aimed to gain a comprehensive understanding of the prevailing landscape of scholarly contributions in the realm of molar distalization within the field of orthodontics. By scrutinizing research articles within related domains, a holistic assessment of the literature was achieved. It was observed that the quantity of publications in this particular discipline remained relatively limited prior to the year 2005. However, since that time, a remarkable surge in scholarly output has been witnessed, culminating in a pinnacle of publication activity in the year 2022.

In regard to the geographic distribution of author affiliations, it is noteworthy that the USA emerges as the most prolific contributor, accounting for the greatest number of published articles. Furthermore, the USA also exhibits the highest centrality score, denoting a heightened degree of collaborative engagement within its scientific community. Conversely, South Korea ranks 2nd in terms of the number of published articles, and 7th in terms of its centrality score, suggesting a comparatively lower prevalence of collaborative research endeavors within its scholarly landscape. In regard to the authors’ institutions, Kyung Hee University has not only published the most literature in this field, but it also has the highest centrality score. The author analysis showed that Park Jae Hyun has achieved the highest publication count. However, it is noteworthy that all authors in this study exhibit relatively low author-centrality scores, indicative of infrequent collaborations across institutions and national borders. The journal analysis showed that the main journals in this field were American Journal of Orthodontics and Dentofacial Orthopedics, which not only publishes the most literature in this field but also has the highest centrality score, which shows the great influence of this journal. The outcomes of the keyword analysis have shed light upon a range of noteworthy research foci within the field. In addition to molar distalization and distal movement, it has become evident that class II malocclusion, anchorage, and the pendulum appliance have emerged as prominent areas of investigation [26]. Furthermore, it is of paramount importance to acknowledge that the focal points of research have evidently evolved over chronological progression. For instance, the initial utilization of external appliances such as headgear and extraoral arch transitioned to the usage of intraoral fixed appliance and micro-screw implant, culminating in the contemporary adoption of clear aligner. This signifies the ceaseless advancement and progression of scholarly research.

The technique of molar distalization primarily finds its application in cases of mild to moderate dental crowding. This approach is most apt for circumstances where there is a reluctance for tooth extraction despite the presence of dental overcrowding. Furthermore, distalization of the maxillary molars is employed to rectify Angle Class II malocclusions [27], whereas the distalization of mandibular molars mitigates Angle Class III malocclusions. Concurrent distalization of both maxillary and mandibular molars offers a solution for both maxillary and mandibular prognathism. At present, the research focus is mainly on the distalization of maxillary molars. The indication for distalisation extends beyond the management of Class II patients, to include Class III surgical patients necessitating decompensation in the upper arch, particularly if the retraction of upper incisors is deemed essential [28]. And the most opportune time to move maxillary first molars distally is before eruption of the second molars [29]. As technological advancements continue to evolve, an increasing number of methods have been introduced to facilitate molar distalisation. Historically, the headgear—an extraoral appliance—has been employed for maxillary molar distalization [30–32]. However, due to its aesthetic unacceptability and the demand for patient compliance, it lacks practicality [30, 33]. As a response to these limitations, intraoral devices such as pendulum, noncompliance intraoral appliances, and distal jet appliances were developed, which do not necessitate patient cooperation [34]. Take noncompliance intraoral appliance as an example, maxillary molar distalization can be effectively performed with the use of noncompliance intraoral appliances [35, 36]. Maxillary first molar distalization ranged from 6.4 to 0.5 mm with a concomitant distal tipping from 18.5° to bodily distalization [35]. A smaller amount of distal movement and a greater amount of crown tipping can be noted at second molars [35]. Nevertheless, these appliances precipitate an inadvertent side effect—the mesial drift of the premolars and incisors, a phenomenon known as anchorage loss [37]. To circumvent this obstacle, the use of intraoral distalization appliances, buttressed by additional miniscrew anchorage is recommended [30, 38, 39]. Moreover, the clear aligner, a method that has garnered immense popularity in recent years [40], has been identified as a significant advancement in this field. The distance of molar distalization is different for each treatment. Based on data from several studies, the pendulum appliances exhibited an average molar distalization ranging from 2 to 6.4 mm [41], with molar distal tipping oscillating between 6.67° to 14.50° [41]. These appliances also instigated anchorage loss, with average premolar and incisor mesial movements measuring from 1.63 to 3.6 mm and 0.9 to 6.5 mm, correspondingly. When analyzing the bone-anchored pendulum appliances (BAPAs) [25], they demonstrated an average molar distalization spanning from 4.8 to 6.4 mm, with distal tipping of molars varying from 9° to 11.3°, and average premolar distalization oscillating between 2.7 to 5.4 mm [41]. The results of molar distalisation were stable in the presented cases 2 years following treatment. The implementation of the distal screw resulted in the attainment of a Class I occlusion of the first molars through a 4.7 mm of distal movement, surpassing the capabilities of traditional appliances [42]. While this process required a longer duration compared to conventional devices, it offered the distinct advantage of a substantial premolar distal movement ranging from approximately 2.1 mm [42]. Clear aligners facilitate the achievement of a remarkable level of precision (88%) in effecting the bodily movement of upper molars [43–47], particularly when a mean distalization movement of 2.7 mm is desired [48]. This accuracy is significantly enhanced through the utilization of attachments. Thus, the utilization of aligners is strongly recommended in cases where non-growing individuals necessitate a range of 2 to 3 mm of distalization in the maxillary molars [48, 49]. In addition, utilizing CBCT imaging [50, 51] and intraoral scanning for diagnostic reasons [52] in conjunction with 3D printing has made possible the fabrication of surgical guides for accurate mini-implant placement [53, 54]. Three-dimensional printing is an additive technology, i.e., a layer-by-layer manufacturing process. In dentistry, 3D printing is used for manufacturing surgical templates, restorations (crowns, inlays, bridges, dentures), and orthodontic appliances [55–57]. Direct 3D printing offers the creation of highly precise clear aligners with soft edges, digitally designed and identically reproduced for an entire set of treatment aligners, offering a better fit, higher efficacy, and reproducibility [56]. These technological advances can provide more options for orthodontists and patients to achieve a win–win and harmonious situation.

## Conclusions

As the tides of time shift and scholars display an ever-growing dedication to unraveling the intricacies of this therapeutic modality, the realm of molar distalization has undergone notable advancements in technology. Initially, the traditional appliance suffered from aesthetic drawbacks and discomfort. However, contemporary iterations of the appliance have transcended these limitations, boasting enhanced elegance and convenience while concurrently elevating their efficacy. Nevertheless, limitations of current appliances, including their durability and propensity for recurrence post-treatment, continue to necessitate further advancement. Hence, the ongoing scientific inquiry aims to delve deeper into refining treatment modalities and fabricating cutting-edge appliances within this realm.

### Advantages and limitations

This investigation boasts numerous laudable characteristics. Fundamentally, it harnesses cutting-edge analytical methodologies to proffer deep-seated perceptions into the advancing trajectories of research across temporal spans, while visually delineating complex networks spanning authors, nations, and scholarly institutions. Furthermore, it transcends traditional metrics habitually harnessed in bibliometric scrutiny, such as impact ratio, H-index, and citation enumerations. In the second instance, the investigation marries automated software scrutiny with rigorous manual inspection of the extant literature, thereby guaranteeing a comprehensive and exacting analysis. This investigation is not without its constraints. One notable limitation of this study pertains to its exclusive dependency on the Web of Science Core Collection as the solitary data source. This reliance may engender an underestimation of the comprehensive body of literature accessible, potentially resulting in the oversight of critical research findings. Moreover, there exists the possibility of bias within the citation data, as papers that garner a high number of citations are not unequivocally synonymous with being the foremost or most precise scientific inquiries.

## Data Availability

Data will be available upon reasonable request.
